# Quantification of clinical scores through physiological recordings in low-responsive patients: a feasibility study

**DOI:** 10.1186/1743-0003-9-30

**Published:** 2012-05-30

**Authors:** Martin Wieser, Lilith Buetler, Heike Vallery, Judith Schaller, Andreas Mayr, Markus Kofler, Leopold Saltuari, Daniel Zutter, Robert Riener

**Affiliations:** 1 Sensory-Motor Systems Lab, Institute of Robotics and Intelligent Systems, Department of Health Science and Technologies, ETH Zurich, Tannenstrasse 1, Zurich, 8092, Switzerland; 2 Medical Faculty, Balgrist University Hospital, University of Zurich, Zurich, Switzerland; 3 HELIOS Clinic Zihlschlacht, Center for Neurological Rehabilitation, Zihlschlacht, Switzerland; 4 Biomedical Engineering, Khalifa University, Abu Dhabi, UAE; 5 Department of Neurology, Hochzirl Hospital, Zirl, Austria; 6 Research Unit for Neurorehabilitation South Tyrol, Bolzano, Italy

**Keywords:** Low-responsive patients, Vegetative state, Minimally conscious state, Clinical score, Quantification, Linear regression

## Abstract

Clinical scores represent the gold standard in characterizing the clinical condition of patients in vegetative or minimally conscious state. However, they suffer from problems of sensitivity, specificity, subjectivity and inter-rater reliability.

In this feasibility study, objective measures including physiological and neurophysiological signals are used to quantify the clinical state of 13 low-responsive patients. A linear regression method was applied in nine patients to obtain fixed regression coefficients for the description of the clinical state. The statistical model was extended and evaluated with four patients of another hospital. A linear mixed models approach was introduced to handle the challenges of data sets obtained from different locations.

Using linear backward regression 12 variables were sufficient to explain 74.4% of the variability in the change of the clinical scores. Variables based on event-related potentials and electrocardiogram account for most of the variability.

These preliminary results are promising considering that this is the first attempt to describe the clinical state of low-responsive patients in such a global and quantitative way. This new model could complement the clinical scores based on objective measurements in order to increase diagnostic reliability. Nevertheless, more patients are necessary to prove the conclusions of a statistical model with 12 variables.

## Background

Defining and detecting “consciousness” is challenging and an area of active discussion in the context of low-responsive patients. The term “consciousness” is classically separated into two components: arousal and awareness [1,2]. Low-responsive patients have their eyes open, exhibit signs of nervous system activation upon stimulation, and therefore show a certain level of arousal. In contrast, they are not aware of themselves and their surroundings, and they show a lack of attention and purposeful behavior, which indicates a low level of awareness. Traditionally, patients in vegetative (VS) and minimally conscious state (MCS) are distinguished by their level of awareness. This means, recovery from a low-responsive stage is detected by observing signs of awareness. However, recent studies have shown that patients who are diagnosed with VS or MCS may retain more awareness than their clinical assessments suggest [3].

Clinical guidelines and scales like the Glasgow Coma Scale (GCS) or the JFK Coma Recovery Scale–revised (JFK CRS-r) represent the gold standard in describing the clinical state of these patients [4-6]. With these clinical methods, diagnoses are mostly based on observable motor behavior. Especially identification of signs of awareness in patients with fluctuating arousal and perceptual, attentional and motor deficits is a great challenge [7]. These clinical assessments rely on conclusions made from motor responses to external stimuli at the time of observation. As a consequence, misdiagnoses of the clinical state in low-responsive patients are common and well-known [8-10].

Because of this unsatisfactory situation new methods to detect and quantify the clinical state of these patients are warranted in order to optimize the treatment as well as the diagnostic decision-making process.

In the past, correlations between autonomic nervous system activity and clinical scores were investigated [11]. Analysis of heart rate (HR), heart rate variability (HRV), electrocardiogram (ECG) frequency bands, as well as skin conductance level showed that recovering from coma is accompanied by an increasing influence of the sympathetic nervous system on HR control and a reintegration of the sympathovagal balance. Dolce et al. [12] suggested that the normalized low-frequency band of the HR plays an important role in residual responsiveness of these patients. Nevertheless, most of these findings showed an interesting trend but not a statistically significance on a group level. Later, correlations between electroencephalography (EEG)-based neurophysiological activity and clinical scores were studied [13-17]. The analysis confirmed that certain cerebral regions are associated with awareness, and that power in the delta band can be considered as a neurophysiological indicator. Furthermore, event-related potentials (e.g. P300 and mismatch negativity) seem to be a viable method to assess residual brain functions and to give evidence on the clinical state without relying on motor behavior [13]. Nevertheless, a general problem of all EEG-based neurophysiological methods is the fact that they have not yet been valid for all etiologies of low-responsive patients, and that there is no universally accepted method to quantify the patients’ clinical state. Recently, neuroimaging techniques, e.g. functional magnetic resonance imaging, became a promising tool in detecting covert signs of awareness. Low-responsive patients showed a reduction of brain metabolism, and during speech/word processing tasks some of the patients demonstrated residual cognitive functions [18-20] or even high-level functions such as learning or active response [15,21-23]. On the one hand, this was the first satisfying proof for a certain level of awareness in these patients. On the other hand, it is still not a universal method to quantify and classify the diversity of low-responsive patients. Further, it is a costly and time-consuming method.

In summary, literature showed that individual pathophysiological signals may contain meaningful information about the clinical state. Nevertheless, a reliable quantification was only possible for specific subgroups of patients. Our new concept is to combine physiological and neurophysiological signals in order to get a more global and robust quantification of the patients’ clinical state. Neurorehabilitation institutions are mostly located outside the large hospital centers, and therefore often lack advanced MRI equipment. In this feasibility study, we focus on a method based on standard clinical equipment that allows examining patients at the bedside. So, there is no need to transfer the patient and in addition, there is no interference between transfer and assessment.

We hypothesize that a combination of traditional clinical physiological signals in a resting condition and neurophysiological signals based on an event-related potential paradigm will help to improve the quantitative description of the clinical state and therefore, complement the clinical scores in an objective way.

## Methods

### Subjects

Thirteen patients (six female and seven male) with average age of 40 years (range: 19-64 years) and average time since onset of injury of 23 months (range: 3–71 months) were included in the study. Patient characteristics are shown in Table 1.

**Table 1:** Overview of all 13 patients

**No.**	**Age, sex**	**Hospital**	**Etiology**	**MSO**	**clinical scores**		
					**GCS**	**JFK CRS-r**	**EFA**
A	45, female	Z	SAH	8	6	2	30
B	64, male	Z	TBI	3	8	4	58
C	59, female	Z	SAH	3	8	7	31
D	50, male	Z	ABI	18	7	6	31
E	49, male	Z	ABI	27	7	5	46
F	20, female	Z	TBI	13	8	7	53
G	44, female	Z	ABI	71	8	6	46
H	34, male	Z	TBI	54	6	7	38
I	52, female	Z	SAH	4	8	2	41
J	19, male	H	TBI	12	3	5	31
K	37, male	H	TBI	17	11	16	47
L	23, female	H	TBI	27	8	8	39
M	24, male	H	TBI	42	8	11	32

MSO: months since onset; GCS: Glasgow Coma Scale; JFK CRS-r: JFK Coma Recovery Scale – revised; EFA: Early Functional Abilities; Z: hospital Zihlschlacht; H: Hochzirl Hospital; TBI: Traumatic Brain Injury; SAH: Subarachnoid Hemorrhage; ABI: Anoxic Brain Injury.

Three patients received beta_1_-selective blockers, but only one patient received a non-selective beta-blocker. Antispastic medication included baclofen in four patients and tizanidine in one. Seven patients received antiepileptic medication, comprising levetiracetam, carabamazepine, lamotrigine, topiramat, valproate, phenytoin, and clonazepam, either alone or in various combinations. One patient each required quetiapine or lorazepam against agitation. Six patients received “activating” medication, either levodopa or amantadine or methylphenidate, while three patients received citalopram. Further, patients were also free of infections requiring antibiotic treatment throughout the study period.

Measurements were conducted between June 2008 and July 2010 at the HELIOS Clinic Zihlschlacht, Switzerland, as well as at Hochzirl Hospital, Austria. The local ethics committees approved the study and a legal representative of each participant gave written informed consent. Task and testing procedures were in accordance with institutional guidelines and the study conformed to the Declaration of Helsinki.

### Experimental protocol

All participants were studied once weekly during an 8 week period. Each measurement session contained one 7-min response period and one 7-min resting period in randomized order. Subjects were in a supine position during the entire experimental session. In the resting period, the physiological measures ECG, respiration signal, galvanic skin response (GSR) and blood pressure (BP) were acquired with a sampling frequency of 1 kHz. In the response period, patients were presented with a classical oddball paradigm and the event-related responses were recorded with a sampling frequency of 1 kHz.

Within 24 h before or following each measurement session, patients were clinically examined and evaluated using the GCS (score: 3–15), JFK CRS-r (score: 0–23) and Early Functional Abilities (EFA) (score: 20–99) [24]. The EFA score was based on a consensus obtained from the treating team of therapists, while GCS and JFK CRS-r were assessed by an external rater. Considering that our patients belong to different locations, there were two external raters, one for each hospital.

### Acquired signals and data analysis

#### Electrocardiogram (ECG)

For the ECG, recordings were obtained from lead I and II of the Einthoven’s triangle with a PowerLab system from AD Instruments, Australia. Signals from the two ECG leads were pre-processed using a 2^nd^ order Butterworth band pass filter of 1–30 Hz. The sum of the two ECG signals was differentiated, and the absolute values of this derivative were compared to an adaptive threshold [25]. Time intervals between heartbeats (R-R intervals) were determined in order to calculate heart rate *HR* as well as standard deviation *S*_*HR*_.

A Poincaré plot, which is a technique known from nonlinear dynamics, allows analyzing fluctuations of the R-R intervals by plotting each R-R interval against the following one. Thus, the relationship between consecutive intervals is graphically displayed in a cloud-like shape known as Poincaré plot. The variable *SD*_*1*_ is defined as the standard deviation of the plot projection onto the negative line of identity, whereas, *SD*_*2*_ is the standard deviation of the projection onto the line of identity. *SD*_*1*_ quantifies the short-term variability and *SD*_*2*_ reflects both short-term and long-term variability. The area of an ellipse *AHRV* with the axis *SD*_*1*_ and *SD*_*2*_ provides general information regarding the HRV [26], an indicator of cardiovascular tone in patients with neurological injuries [27]. Further, Riganello et al. [28] suggested that autonomic changes in VS patients lead to implications to their residual responsiveness.

The sympathetic and parasympathetic balance of the autonomic nervous system is also reflected in the HRV. The Lomb-Scargle method was applied to the series of unevenly sampled R-R intervals in order to perform a spectral analysis [29]. This provides an estimate for the power at specific frequencies. The high-frequency band (HFB) is defined between 0.15 and 0.4 Hz, and power *HFnorm* in this band is normalized to give a total power of 1 from 0.002 to 0.5 Hz. Activity in the HFB appears to derive mainly from vagal activity or the parasympathetic nervous system. The low-frequency band (LFB) is defined between 0.04 and 0.15 Hz, and the normalized activity *LFnorm* is also obtained from division by total power. LFB activity derives both from parasympathetic and sympathetic activity, and it is assumed to reflect the delay in the baroreceptor loop. The ratio of low-to-high frequency spectral power *LF/HF* = *LFnorm*/*HFnorm* is used as an indicator of sympathetic to parasympathetic balance of heart rate fluctuation [26,30].

Additional analysis was based on guidelines for HRV [30]: besides the activity in the HFB and LFB, also the activity *VLF* in the very-low-frequency band between 0.003 and 0.04 Hz was inspected, the square root *RMSSD* of the mean of the sum of the squares as well as the standard deviation *SDNN* of differences between adjacent R-R intervals, the number *NN50count* and percentage *pNN50* of R-R interval differences greater than 50 ms, and the total power *P* in the ECG signal were analyzed.

#### Respiration

A piezoelectric respiratory belt transducer (PowerLab system, AD Instruments, Australia) was used to monitor breathing frequency. This transducer measured changes in thoracic and abdominal circumference during respiration in order to indicate the instances of inhalation and expiration as well as breathing depth. For the given respiration flow signal the peaks (end of inhale), the troughs (end of exhale) and the breathing pauses were identified. So, the average respiration rate *Resp* and the standard deviation *S*_*Resp*_ of the breathing cycles were calculated. Low-responsive patients show respiratory instabilities [31] and therefore, a slow and regular breathing pattern might comprise information about the patient’s physical constitution. The average durations of the inhale and exhale phase, *Tin*_*Resp*_ and *Tex*_*Resp*_^,^ as well as the ratio *κ*_*Resp*_ *= Tin*_*Resp*_*/Tex*_*Resp*_ of the two phases were determined.

#### Galvanic skin response (GSR)

Two bipolar GSR finger electrodes in combination with a GSR amplifier of low voltage and 75 Hz alternating current excitation (PowerLab system, AD Instruments, Australia) were used to obtain the GSR signal. It is known that emotional stimuli could lead to responses in the GSR signal of low-responsive patients and further, the responses are correlated to the clinical state of the patients [32]. According to VaezMousavi et al. [33] arousal and activity are in general reflected in electro dermal activity which can be measured via the skin conductance level. Thus, patients with a higher awareness may also show a different skin response. Specific events within the GSR signal are known as “skin conductance responses”. At these events the response suddenly rises within 4 s to a peak value greater than 0.02μS and then decreases again. The number of detected events *n*_*GSR*_ during the resting period was counted and normalized with the total duration.

#### Blood pressure (BP)

The continuous BP signal was acquired noninvasively by using a CNAP Monitor 500 from CNSystems AG, Austria. Analyzing the BP signal the maxima *BP*_*syst*_ and the minima *BP*_*diast*_ were identified and the estimated values *BP*_*MAP*_ of the mean arterial BP as well as the differences *BP*_*pulse*_ were calculated.

Furthermore, the average duration *Tpeak*_*BP*_ of the BP signal between the occurrence of the minimum and the maximum value (from diastolic BP to the systolic BP value) as well as the duration *Tcycle*_*BP*_ of the whole pulse wave was determined. The BP signal was obtained via a pressure cuff attached to the index and middle finger. Therefore, these values also include information on vessel elasticity. Low-responsive patients belong to a subgroup of the so-called group of bed rest patients. Deconditioning of the patients’ cardiovascular system during immobility is a well-known problem and influences the underlying illness in a negative way [34]. As blood pressure is one of the crucial signals of the cardiovascular system, the clinical state of the patients could also be represented by the introduced parameters.

#### Event-related potentials (ERPs)

Event-related potentials (ERPs) were obtained in a classical oddball paradigm which consisted of 200 stimuli within 400 s; the standard stimulus (500 Hz tone of 100 ms duration) was presented in 85% and the deviant stimulus (1000 Hz tone of 100 ms duration) in 15% of the cases. ERPs seem to be a good method to assess residual brain functions in low responsive patients and may provide evidence on the clinical state without relying on motor behavior [13].

ERPs were obtained from three scalp electrodes using a SynAmps^2^ amplifier system (Compumedics Neuroscan, Germany). Silver-silver-chloride electrodes were used in combination with a SynAmps^2^ Quick-Cap. For reference purposes, 32 electrodes were placed according to the international 10–20 electrode placement standard with an additional reference electrode between Cz and CPz. The electrooculogram was recorded from two pairs of bipolar electrodes: one pair was placed below the outer canthus of each eye, in order to detect horizontal eye movements, the other pair was placed above and below the center of one eye, in order to record vertical eye movements. The software package Scan 4.2 (Compumedics Neuroscan, Germany) was used to record and store the acquired data. Electrode impedance was kept below 10 kΩ. After analog anti- aliasing filtering, data were sampled at 1 kHz and then digitally band-pass filtered, with lower and upper cut-off frequencies of 0.1 and 70 Hz, respectively.

The recorded EEG data were further processed using the BrainVision Analyzer software package from Brainproducts, Germany. EEG data were band-pass filtered from 0.1-12 Hz (Butterworth) and data were re-referenced to an average reference of all 32 electrodes. Ocular artifacts (eye blinks, horizontal eye movements) were corrected applying an independent component analysis (ICA) algorithm provided by the BrainVision software. Thereafter, EEG data were scanned for epochs containing artifacts that exceeded ±100 μV at any electrode and screened visually for further artifacts. All those epochs were excluded from subsequent analysis.

For analyzing individual auditory ERPs the electrodes Fz, Cz and Pz were used. The EEG signal during acoustic stimulation was cut into epochs of 1.5 s including a 100 ms period for baseline correction prior to stimulus onset. Segments were separated and averaged relative to the standard or deviant stimulus. For every electrode position the ERP latency and amplitude were determined. The classical P300 response is expected between 250 and 500 ms after stimulus presentation in healthy subjects [35]. Abnormalities were described in patients with massive cerebral lesions [36,37]. Even for mild brain injuries longer latencies are common [38,39]. Therefore, we extended the criteria for the classical P300 response: in our analysis, we identified a “P300-like” positive peak of the ERP waveform between 250 and 1000 ms as a “late positive event-related response”. Two independent and blinded experts determined the resulting late positive ERP visually and the consensus was accepted for further analysis. The results of these late positive ERPs (amplitudes and latencies) were represented in the variables *Amp*_*Fz*_*, Amp*_*Cz*_*, Amp*_*P*z_*and Lat*_*Fz*_*, Lat*_*Cz*_*, Lat*_*Pz*_. The average amplitude and latency of all three electrodes were defined as *Amp* and *Lat*, respectively. Peak amplitudes of the standard as well as the deviant stimulus were exported and post processed for a subsequent statistical analysis. Analysis of variance (ANOVA) was performed to test the amplitude of the deviant against the standard response. The resulting values representing the significance *Sig*_*Fz*_*Sig*_*Cz*_ and *Sig*_*Pz*_ as well as the average value *Sig* were used as an indication for the distinctiveness of the responses. Further, the classical P300 scalp distribution is defined as the amplitude change over the midline electrodes Fz, Cz and Pz, which typically increases in magnitude from frontal to parietal electrode sites [35]. With regard to this definition, the resulting topographical scalp maps were visually classified into two groups: the “classic group” for scalp maps according to this definition and the “abnormal group” for all the others. The result was captured by the variable *ERP*_*map*_.

### Quantitative description

In a first step data of all patients from hospital Zihlschlacht (*n* = 9) were used to obtain a quantitative description of the clinical state. A linear regression method was used to get fixed regression coefficients for the prediction of the clinical state. In a second step, the statistical model was extended and evaluated with patients at Hochzirl Hospital (*n* = 4). Next to the linear regression method, the linear mixed models approach was introduced to handle the difficulties of data sets at different locations.

### Data preparation

In a first step, tests were performed to prove whether the variables are linearly independent (reference value for the Pearson correlation coefficient < 0.4) and whether the distribution of the independent variables is normal (reference value for skewness/kurtosis between < ±1). Redundant variables were removed and the remaining not normally distributed variables were ln-transformed in order to achieve normal distribution.

Due to different patients’ artifacts (muscle hypertonia, movements, facial expressions, chewing, etc.) and sensor errors, randomly distributed missing variables occurred. Rubin [40] and others have demonstrated the disadvantage of simply deleting cases with missing data. They argued that even with a small number of imputations, estimation quality improves strongly. Therefore, we used the analyzed data to predict missing data via a multiple regression method. As suggested by Rubin, for each estimated value the residual of a randomly picked case was added in order to avoid an unrealistically low level of noise.

Finally, a backward method was used, starting with all remaining independent normally distributed variables, and reducing the set of variables stepwise according to the criterion: probability of F-to-remove ≥ 0.1. For all statistical calculations the software package SPSS 17 (IBM Corporation, USA) was used.

### Linear regression

A linear regression was used to obtain a quantitative description of the clinical state. Linear regression analysis estimates the coefficients of a linear equation, involving several independent variables that best predict the value of a dependent variable. As the dependent variable, the average of the two clinical scales JFK CRS-r and EFA was used. For that purpose, both scales were normalized (normalized range: 0 to 100) in order to average the relative change of the scores. All variables *x*_*i*_ regarding the analysis of the physiological and neurophysiological signals were used as independent variables:

(1) $$Score{\;}_{\mathrm{clinical}}=\beta_{0}+\sum_{i=1}^{n}\beta_{i}x_{i}+\epsilon$$

with *β*_0_: intercept, *β*_i_: regression coefficients, *ϵ*: error term and *n*: number of used variables. The acceptability of the model as well as the goodness of fit were tested with an ANOVA and the R-squared value and the pattern of residuals was inspected.

In our study we investigated only chronic patients (months since onset ≥ 3) in order to reach a more homogeneous group of patients. The goal of the presented method was an individual modeling of the patients’ clinical state that is based on objective measurements without relying on the time of onset. Therefore, the development of the clinical state of every single patient was the focus of the method and the variable “months since onset” was not used for further analysis.

### Linear mixed models

The original linear model had one random effect, the error term *ϵ*. All the other regression coefficients *β*_*i*_ were fixed. In our case, they were fixed for all patients to enable the estimation of the clinical state for future patients. In contrast, linear mixed models include additional random effects in order to provide more degrees of freedom for the model. Using the quantitative description for different hospitals, a single random effect (the error term *ϵ*) could not explain the additional effects introduced by the different locations as well as different background, expertise and experience of the therapeutic teams. To evaluate the quantification method, the estimation of the clinical state of patients from Hochzirl Hospital was done with the linear regression as well as with the linear mixed model approach with one additional random effect. Finally, the results of both models were compared to each other.

## Results

After testing normal distribution and cross correlation of all presented 39 variables only 19 variables remained. Seven out of these 19 were ln-transformed in order to reach a normal distribution (see Additional file 1). Linear backward regression was applied to the variables and the R-squared was maximal with 12 variables (Table 2, Additional file 1). The ranking of the variables in Table 2 was based on their standardized coefficients. The proportion of variation in the dependent variable explained by the regression model is expressed by the value of the R-squared of 0.744.

**Table 2:** Results of the linear backward regression

**Rank**	**ECG**	**ERP**	**BP**	**Respiration**
1		^*ln(Lat*^*Pz*^*)*^		
2		*Sig*_*Cz*_		
3	*ln(LFnorm)*			
4	*ln(A*_*HRV*_*)*			
5				*ln(κ*_*Resp*_*)*
6			*BP*_*MAP*_	
7				*Resp*
8			*BP*_*pulse*_	
9		*Amp*_*Fz*_		
10	*VLF*			
11		*Lat*_*Cz*_		
12			*ln(Tpeak*_*BP*_*)*	

Ranking of the 12 variables based on the linear regression method.

**Figure 1:**
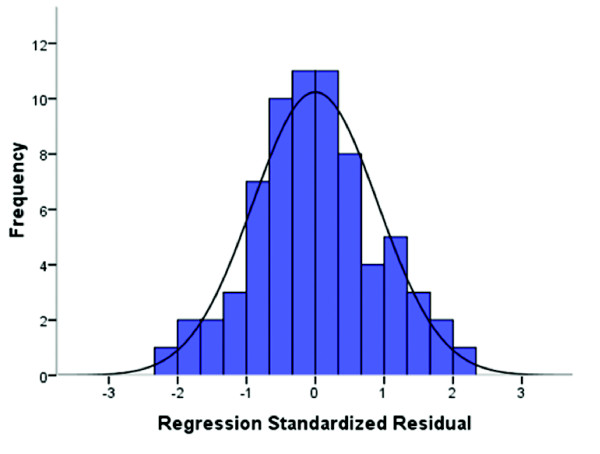
**Evaluation of the regression model.** Distribution of the standardized residuals regarding the linear regression model. In solid black a normal distribution density function is shown

Further, an ANOVA was performed to test the acceptability of the model from a statistical point of view. The null hypothesis that all population values for the regression coefficients are zero could be rejected (*F* = 13.78 and *p* < 0.001). In order to evaluate the quality of the regression model, the distribution of standardized residuals is illustrated superimposed with a normal distribution density function Figure 1 shows the histogram of all standardized residuals originating from single measurement sessions.

**Figure 2:**
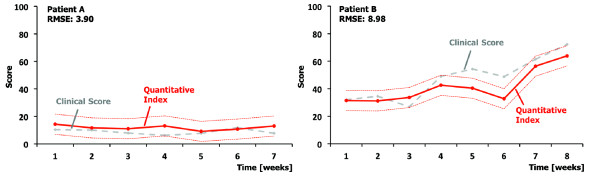
**Results of the regression model.** The clinical score (grey, dashed line) and the quantitative index (red, solid line) with the standard error (red, dotted line) of two single patients over a period of several weeks.

The normalized change of the clinical scores compared to the used model containing 12 variables is shown in Figure 2. Data are based on two single patients over a period of several weeks. From a clinical point of view, the first patient (Figure 2, left side) was very stable, whereas, the second patient (Figure 2, right side) showed a 40% improvement on the clinical score. The result of the regression model is shown as quantitative index with a 7.30 standard error of the estimate (Figure 2, solid and dotted red lines). The root mean square error (RMSE) was 3.90 for the first patient and 8.98 for the second patient. The average RMSE of all nine patients of the hospital Zihlschlacht was 6.4 (range: 3.90 – 8.98; see also Additional file 2). Transformed back to the clinical scores, this value would correspond to 1.5 points on the JFK CRS-r and 5.1 points on the EFA score. All nine patients from hospital Zihlschlacht are included in this modeling process and, therefore, in the first step of evaluation we included only eight patients for the modeling process and used the result to estimate the clinical score of the last one (leave-one-out cross- validation method). Performing this method for all nine patients separately, the average RMSE in the evaluation process was increased to 10.35 (range: 6.21–14.67).

**Figure 3:**
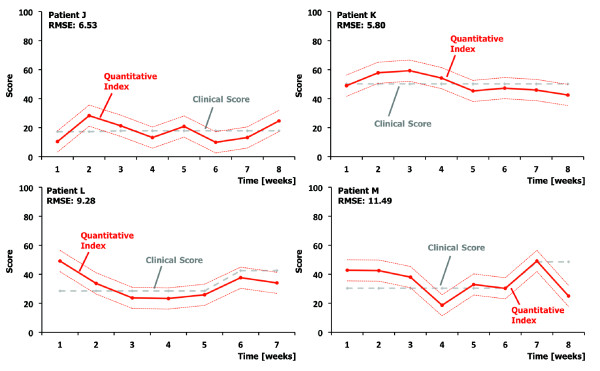
**Results of the linear mixed model.** Evaluation of the model applied for the patients at Hochzirl Hospital. The clinical score (grey, dashed line) and the quantitative index (red, solid line) with the standard error (red, dotted line).

To extend the linear regression model to an extended group of low-responsive patients, the model was also used to estimate clinical scores at Hochzirl Hospital. The results of all four patients at Hochzirl Hospital were analyzed and the generalization of the model led to an averaged RMSE of 12.30 (range: 6.77–17.54). In order to deal with the offset (average offset: 6.84) resulting from the fixed linear regression model, a linear mixed model was introduced. The intercept was extended with one additional random effect and the resulting RMSE dropped to 8.28 (range: 5.80–11.49). This average value corresponds to 1.9 points on the JFK CRS-r as well as 6.5 points on the EFA score. The final results of all four patients at Hochzirl Hospital are depicted in Figure 3.

## Discussion

### Clinical scores as reference

Clinical scores are the gold standard to assess low-responsive patients, yet they suffer from low sensitivity, specificity, and inter-rater reliability, and above all from high subjectivity and rater’s experience. Some of the discrepancies in the different local trends of the scores may be attributed to certain clinical conditions when patients emerging from long-lasting states of unresponsiveness attain a certain level of vigilance but at the same time develop a stage of fluctuating agitation, aggression, and negativism. In such a condition patients are awake, so that functional abilities are theoretically present, but they make hardly use of their gaining functions. Such a situation, of course, sets the stage also for discrepancies in the evaluation of a patient’s functional abilities.

To address this problem, we used two different clinical scores (JFK CRS-r and EFA) and took the average of the normalized change as our dependent variable, which reduces uncertainty of the single score values. On one hand, dedicated external raters for each hospital examined the JFK CRS-r, providing a neutral “snap-shot” of a patient’s clinical state at that particular time point. On the other hand, a team of therapists who had worked with the patients over a period of days examined the EFA. Consequently, the EFA is an expression of the patient’s clinical state over the last days. In general the EFA focuses more on the functional abilities and the JFK CRS-r more on the coma recovery status of the patient. Both scores are suitable to describe the clinical state of patients in a VS or MCS. All patients in the present study were either vegetative or minimally conscious, hence, the two scores were able to describe all stages of our patients without ceiling effect. The combination of both ratings leads to a broad and robust expression of the clinical state and was therefore used as dependent variable for our regression model. Because of the linear combination of both clinical scores, one point on the new quantitative index corresponds to 0.23 points on the JFK CRS-r and 0.79 points on the EFA score.

### Evaluation of the quantitative description

In a first evaluation step only nine patients from the hospital Zihlschlacht were used. Via the described leave-one-out cross validation method the average RMSE increased about 3.95. This increase of the value was expected because the total number of patients is quite low. Consequently, leaving one patient out for evaluation reasons, over 11% of the measured data could not be used for the model.

In the second evaluation step all nine patients were used for the regression model and the residual four patients measured at Hochzirl Hospital were used for evaluation. Due to their background, expertise, experience, and different patient mix in the respective environment, the way how different therapeutic teams reach their decision how to score a given patient at a certain time may slightly differ among hospitals, contributing to the offset between hospitals. To cope with these challenges, the linear mixed model was introduced. The existing linear regression model was extended with one random effect regarding the intercept. So, the relative development of the estimation is not effected using the linear mixed model approach. The occurred average offset without the additional random effect was 6.84. This constant change of the estimation could be adjusted with the modified intercept.

The estimation quality for the first two patients at Hochzirl Hospital, J and K, is as good as for the patients from the hospital Zihlschlacht. There is an outlier in the first week, but in general the estimation of patient L is also satisfactory. Looking at the last patient M, the general estimation was weak, nevertheless, the estimation of four weeks stays within the standard error of the estimate.

Finally, there is the possibility that nonlinear methods may lead to a further improvement of the results. In this work we did not evaluate nonlinear methods because to our knowledge there is no evidence in the literature how the presented variables are related to the clinical state of low-responsive patients. In general there are only trends and indications for such possible relationships. We therefore decided to use a linear combination of all variables, because the results are easier to interpret. Furthermore, data from 13 patients are statistically too weak in order to make sound conclusions regarding significant nonlinear relationships.

### Importance of individual variables

To determine the importance of individual variables the standardized coefficients were evaluated (see Additional file 1). The ERP variables *Lat*_*Pz*_ and *Sig*_*Cz*_ as well as the remaining ECG variables *LFnorm* and *A*_*HRV*_ had the highest impact in the linear regression method (−1.00, 0.43,–0.35,–0.35, respectively). Only few patients received medication that could potentially influence the ECG: betablockers (*n* = 4) and phenytoin (*n* = 1). ERPs are mostly influenced by vigilance. Despite a potentially sedating effect of some of our medication (antispastic, antiepileptic, or neuroleptic medication) none of our patients was drowsy or fell asleep during the measurements. Therefore, in this model, variables regarding ERPs and ECG contribute most to the description of the clinical scores. One of the final goals is the reduction of the amount of measurements while sustaining the quality of the quantitative description. It seems that the ERPs as well as the ECG signals are crucial measurements, whereas, the GSR signal is less important. To confirm this assumption data from more patients will be necessary.

Furthermore, the entity of all 12 variables has a high impact for estimating the clinical state. The analysis showed that information of individual variables is not robust and their interpretation in terms of the clinical state of the patient could be weak. This confirms findings from other research groups [11,12,14-17]. However, the combination of all 12 variables showed that the ensemble could formulate a stronger conclusion. The results are quite promising and to our knowledge this is the first attempt to describe the clinical state of low-responsive patient in such a general quantitative way. From a statistical point of view, the acceptability of the model as well as the distribution of residuals (Figure 1) was satisfactory. So, with the current study the feasibility of a quantitative description could be shown.

### Clinical relevance

Monti et al. [3] pointed out that current clinical diagnostic methods are limited and guidelines should be modified to include independent sources of diagnostically relevant information.

This new method has the potential to objectify the patient’s’ clinical state and improve inter-rater variability. In addition to the qualitatively identified clinical score, this new objective-measurement based model of the patients’ clinical state will complement the clinical statement. Thus, patient assessments as well as the relative development of the clinical state could be rendered more comparable between different hospitals, and treatment outcomes could be monitored in a more quantitative way.

### Limitations of the study

The findings of this feasibility study are quite promising despite the limitation that data for the model are based upon nine patients only with varied medical conditions, which is typical for this kind of population. More patients are necessary to proof the conclusions of this statistical model with 12 independent variables. Especially the identification of signs of awareness in patients with fluctuating arousal and perceptual, attentional and motor deficits is a great challenge [7]. This requires additional measurements with equal patient groups for different etiologies (TBI, SAH and ABI). Clinical assessment relies totally, and the quantitative measurements partly on responses to external stimuli that are observed at the time of examination. The JFK CRS-r was assessed within 24 hours, and not simultaneously with the quantitative measurements, which may cause an additional error in the model. Nevertheless, for the regression model the JFK CRS-r was combined with the EFA score and this score is an expression of the patient’s clinical state over the last days. Consequently, a potential error of the JFK CRS-r regarding timing could be partly compensated.

Patients in VS or MCS often require tracheostomy, which might alter the breathing pattern since it reduces work of breathing muscles. However, only three patients from hospital Zihlschlacht, but none from Hochzirl Hospital, had a tracheostomy with a canula in place. Two of them were in the process of weaning during the time of the study, suggesting that their breathing was already being trained for physiological conditions. Hence, the influence of altered breathing on our results seems to be negligible.

## Conclusion & outlook

Using linear backward regression, 12 variables were sufficient to explain 74.4% of the variability in the change of the clinical scores JFK CRS-r and EFA. Variables based on ERPs and ECG account for most of the variability. Altogether, the results are quite promising considering that this is the first attempt to describe the clinical state of low-responsive patients in such a global and quantitative way.

More patient data and additional measures will enable refinement of the methods and additional classifications to be incorporated into the new objective-measurement based model of the clinical state. Furthermore, additional evaluation measurements in more hospitals should be applied to improve and elucidate the data for applications in a clinical context. To strengthen the objectivity of the method, neuroimaging techniques like fMRI could be included and evaluated in future studies.

The project vision would be to develop a possibility to distinguish between different clinical stages of low-responsive patients via an objective measurement. This would improve the quality of diagnosis and reduce the frequency of misdiagnoses. Finally, the ability to prognosticate the rehabilitation development would satisfy a major request of many rehabilitation hospitals.

## Abbreviations

VS, Vegetative state; MCS, Minimally conscious state; GCS, Glasgow Coma Scale; JFK CRS-r, JFK Coma Recovery Scale – revised; HR, Heart rate; HRV, Heart rate variability; ECG, Electrocardiogram; EEG, Electroencephalography; MMN, Mismatch negativity; TBI, Traumatic Brain Injury; SAH, Subarachnoid Hemorrhage; ABI, Anoxic Brain Injury; GSR, Galvanic skin response; BP, Blood pressure; EFA, Early Functional Abilities; HFB, High-frequency band; LFB, Low-frequency band; ERP, Event-related potential; ICA, Independent component analysis; ANOVA, Analysis of variance; RMSE, Root mean square error.

## Competing interests

The authors declare that they have no competing interests.

## Authors’ contribution

MW, LB, AM, MK, LS, DZ and RR participated in the design of the study. MW, LB, JS, AM and MK conducted the recordings in the hospitals and MW, LB and HV carried out the data analysis and drafted the manuscript. RR initiated the study and took the responsibility for the organization. All authors read and approved the final manuscript.

## Supplementary Material

Additional file 1 **Overview of all normal distributed and linearly independent variables.** Linear backward regression was applied to the variables and R-squared was maximal with 12 variables. All 12 variables are listed in the table together with the standardized as well as non-standardized regression coefficients and standard errors. The coefficients are part of the equation in the linear regression section. **Results of the regression model.** The clinical score (grey, dashed line) and the quantitative index (red, solid line) with the standard error (red, dotted line) of patient C to I. Click here for file

Additional file 2 **Results of the regression model.** The clinical score (grey, dashed line) and the quantitative index (red, solid line) with the standard error (red, dotted line) of patient C to I. Click here for file
