# Supplementary material for: Quantification of clinical scores through physiological recordings in low-responsive patients: a feasibility study
Source: J Neuroeng Rehabil. 2012 May 30;9:30. doi: 10.1186/1743-0003-9-30 (PMC3443429; doi:10.1186/1743-0003-9-30)
Supplement: Additional file 1 — Overview of all normal distributed and linearly independent variables. Linear backward regression was applied to the variables and R-squared was maximal with 12 variables. All 12 variables are listed in the table together with the standardized as well as non-standardized regression coefficients and standard errors. The coefficients are part of the equation in the linear regression section. Results of the regression model. The clinical score (grey, dashed line) and the quantitative index (red, solid line) with the standard error (red, dotted line) of patient C to I. [file 1743-0003-9-30-S1.pdf]

Additional file 1 Overview of all normal distributed and linearly independent variables. Linear backward regression was applied to the variables and R-squared was maximal with 12 variables. All 12 variables are listed in the table together with the standardized as well as non-standardized regression coefficients and standard errors. The coefficients are part of the equation in the linear regression section

no.	variables	selection (ranking)	standardized coefficient	non-standardized coefficient	
			β_{norm}	$\beta_{1\text{-}12}$	SE
1	$\ln(A_{HRV})$	4	-0.353	-2.412	0.672
2	$\ln(LFnorm)$	3	-0.353	-6.968	1.761
3	VLF	10	0.234	0.018	0.007
4	$Resp$	7	0.337	0.841	0.242
5	$\ln(S_{Resp})$	---			
6	$\ln(\kappa_{Resp})$	5	-0.344	-9.650	2.097
7	$\ln(n_{GSR})$	---			
8	BP_{MAP}	6	-0.339	-0.359	0.088
9	BP_{pulse}	8	0.328	0.329	0.107
10	$\ln(Tpeak_{BP})$	12	0.140	8.104	4.441
11	$Tcycle_{BP}$	---			
12	Amp_{Fz}	9	-0.293	-1.778	0.518
13	Amp_{Pz}	---			
14	Lat_{Fz}	---			
15	Lat_{Cz}	11	0.212	0.013	0.005
16	$\ln(Lat_{Pz})$	1	-1.000	-20.525	2.245
17	Sig_{Fz}	---			
18	Sig_{Cz}	2	0.426	4.212	0.855
19	Sig_{Pz}	---			
