# Supplementary material for: Quantification of clinical scores through physiological recordings in low-responsive patients: a feasibility study
Source: J Neuroeng Rehabil. 2012 May 30;9:30. doi: 10.1186/1743-0003-9-30 (PMC3443429; doi:10.1186/1743-0003-9-30)

Additional file 2 Results of the regression model. The clinical score (grey, dashed line) and the quantitative index (red, solid line) with the standard error (red, dotted line) of patient C to I

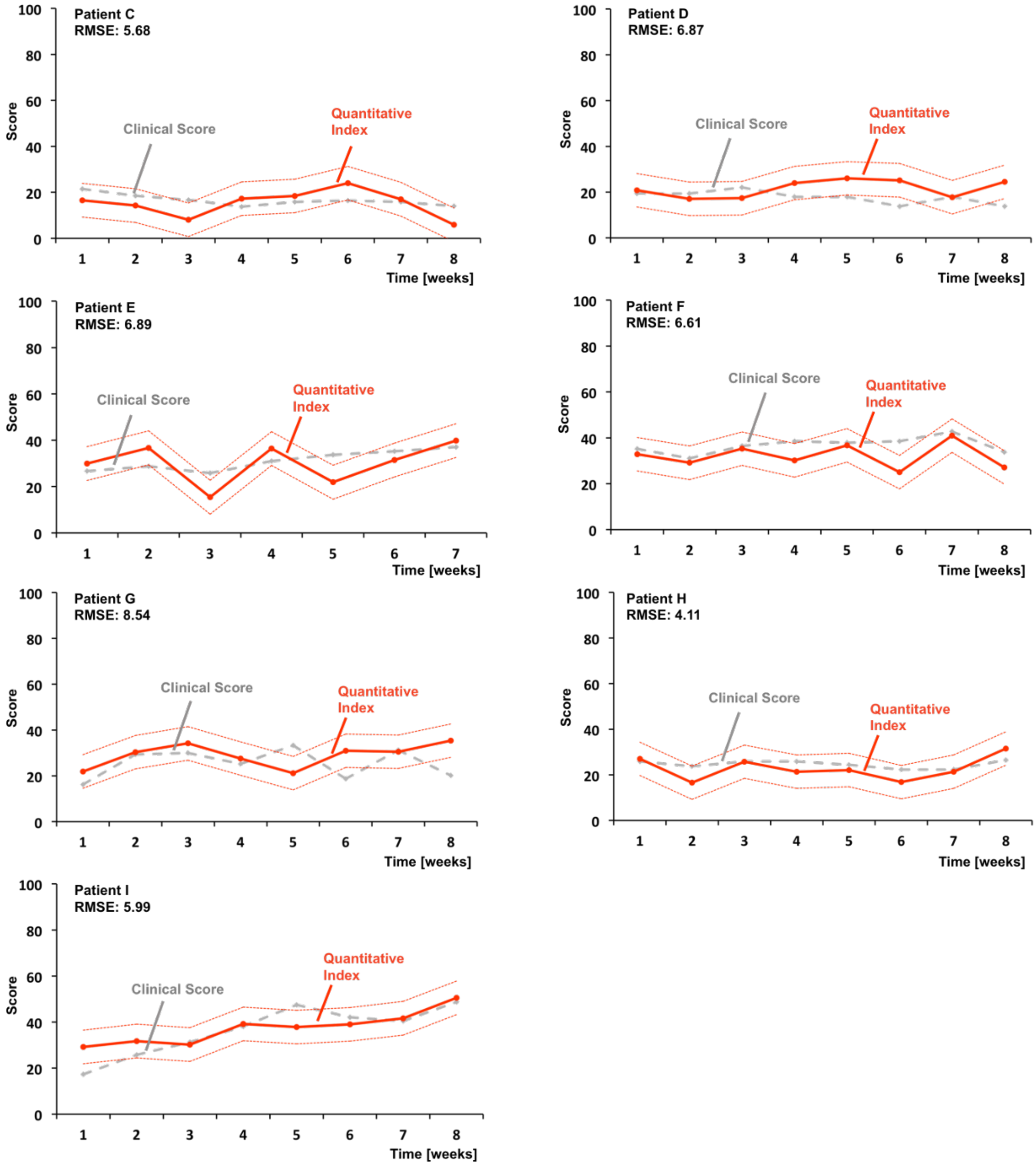

Supplement: Additional file 2 — Results of the regression model. The clinical score (grey, dashed line) and the quantitative index (red, solid line) with the standard error (red, dotted line) of patient C to I. [file 1743-0003-9-30-S2.pdf]
